# Diagnosis and Management of Mixed Gestational Trophoblastic Neoplasia: A Study of 16 Cases and a Review of the Literature

**DOI:** 10.3389/fonc.2019.01262

**Published:** 2019-11-15

**Authors:** Yujia Kong, Guangshi Tao, Liju Zong, Junjun Yang, Xirun Wan, Wenze Wang, Yang Xiang

**Affiliations:** ^1^Department of Obstetrics and Gynecology, Peking Union Medical College Hospital, Chinese Academy of Medical Sciences & Peking Union Medical College, Beijing, China; ^2^Department of Obstetrics and Gynecology, The Second Xiangya Hospital of Central South University, Changsha, China; ^3^Department of Pathology, Peking Union Medical College Hospital, Chinese Academy of Medical Sciences & Peking Union Medical College, Beijing, China

**Keywords:** choriocarcinoma, placental site trophoblastic tumor, epithelioid trophoblastic tumor, diagnosis, treatment

## Abstract

**Objective:** Mixed gestational trophoblastic neoplasia (GTN) is a rare occurrence that refers to the coexistence of choriocarcinoma and/or placental site trophoblastic tumor and/or epithelioid trophoblastic tumor. The diagnosis and management of mixed GTN are challenging. We investigated the clinicopathological characteristics, diagnoses, treatments, and outcomes of patients with mixed GTN.

**Materials and Methods:** The medical records and pathological sections of 16 patients with mixed GTN who were treated at Peking Union Medical College Hospital and The Second Xiangya Hospital of Central South University between January 2012 and December 2018 were reviewed.

**Results:** Pretreatment serum human chorionic gonadotropin (hCG) levels ranged from 180 to 625,024 IU/L, and were >10,000 IU/L in 14 of the 16 patients, none of whom were diagnosed correctly at initial presentation. Two patients were diagnosed with choriocarcinoma coexisting with intermediate trophoblastic tumor (ITT) through dilation and curettage (D&C) before treatment. Another 5 patients were histologically confirmed to have placental site trophoblastic tumor (PSTT) by D&C but final pathological findings showed mixed PSTT and choriocarcinoma at subsequent hysterectomy. Seven post-chemotherapy patients with an initial clinical diagnosis of choriocarcinoma underwent surgery because of chemoresistance and their pathological findings revealed coexisting ITT. The remaining 2 patients were found to have choriocarcinoma coexisting with ITT following cervical biopsy and pulmonary lobectomy. All patients received chemotherapy: 14 underwent surgery combined with chemotherapy and 2 received chemotherapy alone to preserve fertility. Other than 1 patient who died of disease progression, 15 patients (93.8%) achieved complete remission (CR) after treatment, although 5 (33.3%) relapsed. Of these 5 patients with relapse, 3 achieved CR after additional treatment, 1 was receiving an immune checkpoint inhibitor, and 1 was lost to follow-up after refusing further therapy.

**Conclusion:** Mixed GTN is difficult to diagnose on initial presentation. Overlap of the ITT component should be considered in refractory chemoresistant choriocarcinoma. Coexistence of choriocarcinoma should be suspected in ITT patients with high hCG levels. Surgery combined with chemotherapy is optimal treatment for choriocarcinoma mixed with ITT.

## Introduction

Gestational trophoblastic neoplasia (GTN) refers to a group of uncommon malignant gynecological tumors, including invasive mole, choriocarcinoma, and intermediate trophoblastic tumor (ITT). Specifically, ITT comprise placental site trophoblastic tumor (PSTT) and epithelioid trophoblastic tumor (ETT) (1). Although all choriocarcinoma, PSTT, and ETT arise from abnormal trophoblast proliferation, the diagnosis and management of choriocarcinoma totally differ from those of PSTT or ETT. Choriocarcinoma can be diagnosed based on clinical presentation and markedly elevated serum human chorionic gonadotropin (hCG); therefore, histological evaluation is generally not required for its diagnosis. The first-line treatment for choriocarcinoma is chemotherapy alone, which is sufficient to cure the majority of patients (2). However, PSTT and ETT are rare types of GTN that are diagnosed based on specific pathological evidence. In contrast to patients with choriocarcinoma, serum hCG levels in patients with PSTT and ETT are often normal or only mildly elevated owing to the absence of syncytiotrophoblast (3). Both PSTT and ETT are less sensitive to chemotherapy than choriocarcinoma; hence, hysterectomy is the primary treatment recommended for these tumors without metastases (2–4). Additionally, chemotherapy should be considered for patients with metastases and/or those with high-risk prognostic factors (4–6).

However, some patients may have mixed GTN, which comprise choriocarcinoma and/or PSTT and/or ETT. Such mixed GTNs can be suspected by dilation and curettage (D&C) before treatment; otherwise, some patients with initially clinical diagnosis of choriocarcinoma undergo post-chemotherapy surgery owing to chemoresistance, whereupon postoperative pathological evidence reveals coexisting ITT (thereby explaining the resistance to chemotherapy). However, owing to their rarity, mixed GTNs have mostly been described in case reports to date (7–19), and limited information exists regarding mixed GTN, making their diagnosis and management challenging.

Therefore, we conducted this retrospective study to review a series of patients with mixed GTN treated at 2 Chinese medical centers. To the best of our knowledge, this study contained the largest sample size of patients with mixed GTN investigated to date. We highlight their clinicopathological characteristics, diagnoses, treatments, and outcomes for the first time, and provide further information concerning this rare entity.

## Materials and Methods

We conducted a retrospective review of all patients with GTN who were treated at Peking Union Medical College Hospital (PUMCH) Trophoblastic Disease Center and The Second Xiangya Hospital of Central South University from January 2012 to December 2018. Mixed GTNs referred to patients with components of choriocarcinoma and/or PSTT and/or ETT. A total of 16 patients diagnosed with mixed GTN were identified, including 13 from PUMCH and 3 from The Second Xiangya Hospital of Central South University. All 16 patients were confirmed to have choriocarcinoma coexisting with PSTT or ETT upon examination of their pathological sections by gynecologic pathologists. The database and patients' medical records were reviewed to extract the age at diagnosis, antecedent pregnancy, interval between last pregnancy and treatment, presenting symptoms, pre- and post-treatment serum hCG levels, initial diagnosis, pathological diagnosis, International Federation of Gynecology and Obstetrics stage and risk score, treatment, and survival information. All patients provided written consent to using their record data and portions of their surgical specimens for scientific research. This study was performed with the approval of the ethics committees of both participating institutions.

Treatment efficiency was evaluated based on 3 parameters: complete remission (CR), resistance, and relapse. CR referred to normalization of serum hCG for at least 4 consecutive weeks; resistance was considered serum hCG reaching a plateau or increasing after 2–3 courses of chemotherapy; relapse was defined hCG levels rising again after CR.

The patients were followed based on our institutional standards. Serum hCG levels were measured weekly for 1 month, monthly during the following 1 year, followed by every 3 months for the second year, then every 6 months until 5 years and finally once a year afterwards.

## Results

### Clinical Characteristics

The clinical characteristics of the 16 patients with mixed GTN are summarized in Table 1. The mean age at diagnosis was 33.5 years (range, 22–50 years). Abnormal vaginal bleeding was the most common symptom in 14 patients. Moreover, one patient presented with persistent elevated serum hCG after an abortion and another presented with pulmonary nodules. Antecedent pregnancies were completed to term in 7 patients (43.8%) and ended in abortion in 9 (56.2%). None of the patients had an antecedent molar pregnancy. The interval between the antecedent pregnancy and treatment was ≤12 months for 8 patients and >12 months in the remaining 8 (50.0% each). Pre-treatment serum hCG levels ranged from 180 to 625,024 IU/L. Notably, 14 of 16 patients had high levels of hCG >10,000 IU/L. The majority of patients presented with uterine lesions, although 2 (patients #13 and #16) did not have uterine lesions throughout the course of disease. The size of uterine lesions ranged from 2.5 to 7.0 cm. The tumor was confined to the uterus in 6 patients (stage I), 1 patient had vaginal metastases (stage II), 7 patients had lung metastases (stage III), and 2 patients had brain or liver metastases (stage IV).

**Table 1:** Characteristics, diagnoses, and treatments of patients with mixed gestational trophoblastic neoplasia in our cohort.

**Patients No**.	**Age**	**Symptoms**	**Antecedent pregnancy**	**Pretreatment hCG (IU/L)**	**FIGO stage**	**Initial presumptive diagnosis**	**Diagnostic method**	**Pathological diagnosis**	**Treatment**	**Relapse**
1	24	Abnormal vaginal bleeding	Abortion	32,980	I	CC	D&C	CC/PSTT	7 cycles of FAEV	No
2	30	Abnormal vaginal bleeding	Term	625,924	I	CC	D&C	CC/ETT	6 cycles of FAEV	No
3	30	Abnormal vaginal bleeding	Term	152,580	III	CC	D&C and TAH+BS	CC/PSTT	2 cycles of FAEV, TAH+BS, then 3 cycles of FAEV	No
4	41	Abnormal vaginal bleeding	Abortion	372,610	I	CC	D&C and TAH+BS	CC/PSTT	3 cycles of FAEV, TAH+BS, then 5 cycles of FAEV	No
5	50	Abnormal vaginal bleeding	Abortion	36,747	III	CC	D&C and LH+BS	CC/PSTT	3 cycles of FAEV, LH+BS, then 1 cycle of FAEV	No
6	33	Elevated hCG after abortion	Abortion	29,000	III	CC	D&C and LH+BS	CC/PSTT	3 cycles of EMA/CO, LH+BS, then 3 cycles of EMA/CO	No
7	28	Abnormal vaginal bleeding	Abortion	10,960	I	CC	D&C and LH+BS	CC/PSTT	4 cycles of FAEV, LH+BS, then 3 cycle of FAEV	No
8	44	Abnormal vaginal bleeding	Term	234,916	I	CC	Resection of tumor	CC/PSTT	5 cycles of FAEV, resection of uterine lesion, then 2 cycles of FAEV	No
9	25	Abnormal vaginal bleeding	Abortion	16,431	III	CC	LH+BS	CC/ETT	4 cycles of FAEV, 3 cycles of EMA/EP, LH+BS, then 4 cycles of EMA/EP	Yes
10	22	Abnormal vaginal bleeding	Abortion	26,410	III	CC	TAH+BS	CC/PSTT	2 cycles of EMA/CO, 1 cycle of FAEV, 3 cycles of EMA/EP, TAH+BS, then 3 cycles of EMA/EP	No
11	36	Abnormal vaginal bleeding	Term	60,945	III	CC	LH+BS	CC/ETT	6 cycles of FAEV, 4 cycles of EMA/CO, LH+BS, then 1 cycle of FAEV, and pulmonary lobectomy	Yes
12	24	Abnormal vaginal bleeding	Term	6,747	II	CC	LRH+BS	CC/ETT	2 cycles of FAEV, 3 cycles of EMA/CO, 3 cycles of EMA/EP, LRH+BS, then 4 cycles of EMA/EP	Yes
13	36	Abnormal vaginal bleeding	Abortion	96,750	IV	CC	Pulmonary lobectomy	CC/ETT	4 cycles of EMA/CO, 2 cycles of FAEV, 4 cycles of EMA/EP, 2 cycles of TE/TP, pulmonary lobectomy, and then 2 cycles of TE/TP	No
14	42	Abnormal vaginal bleeding	Term	143,484	IV	CC	LH+BS	CC/ETT	9 cycles of EMA/CO, 3 cycles of EMA/EP, 5 cycles of TE/TP, LH+BS, then 4 cycles of TE/TP	Died
15	42	Abnormal vaginal bleeding	Term	17,425	I	Cervical cancer	Cervical biopsy and LH+BS	CC/ETT	2 cycles of FAEV, LH+BS, then 3 cycles of FAEV	Yes
16	30	Pulmonary nodules	Abortion	180	III	Lung cancer	Pulmonary lobectomy	CC/ETT	Pulmonary lobectomy, and then 3 cycles of FAEV	Yes

*CC, choriocarcinoma; PSTT, placental site trophoblastic tumor; ETT, epithelioid trophoblastic tumor; D&C, dilation and curettage; LH, laparoscopic hysterectomy; TAH, total abdominal hysterectomy; LRH, laparoscopic radical hysterectomy; BS, bilateral salpingectomy; FAEV, floxuridine, actinomycin-D, etoposide, vincristine; EMA/CO, etoposide, methotrexate, actinomycin-D/cyclophosphamide, vincristine; EMA/EP, etoposide, methotrexate, actinomycin-D/etoposide, cisplatin; TE/TP, paclitaxel, etoposide/paclitaxel, cisplatin*.

### Diagnostic Evaluation

The initial diagnoses of the patients with mixed GTN prior to pathological evaluation were suspected choriocarcinoma in 14, cervical cancer in 1, and lung cancer in 1. Among the 16 patients, 2 patients (#1–2) underwent D&C owing to irregular vaginal bleeding, whereupon pathological findings revealed choriocarcinoma coexisting with ITT. Another 5 patients (#3–7) were histologically confirmed to have PSTT by D&C. Subsequently, hysterectomy was performed in these patients and the final pathological findings showed PSTT coexisting with choriocarcinoma. Moreover, 7 patients (#8–14) received chemotherapy as their primary treatment owing to the clinical diagnosis of choriocarcinoma without pathological evidence. However, all of them ultimately underwent hysterectomy or pulmonary lobectomy because of chemoresistance or persistent tumor lesions and the postoperative pathological findings revealed choriocarcinoma coexisting with ITT. One patient (#15) who presented with a cervical mass underwent a cervical biopsy; her pathological findings was suspected of choriocarcinoma mixed with ETT which was confirmed by pathological samples post-hysterectomy. Another patient (#16) who was initially suspected of having lung cancer underwent pulmonary lobectomy and was pathologically diagnosed with choriocarcinoma coexisting with ETT.

### Pathological Features

**Figure 1:**
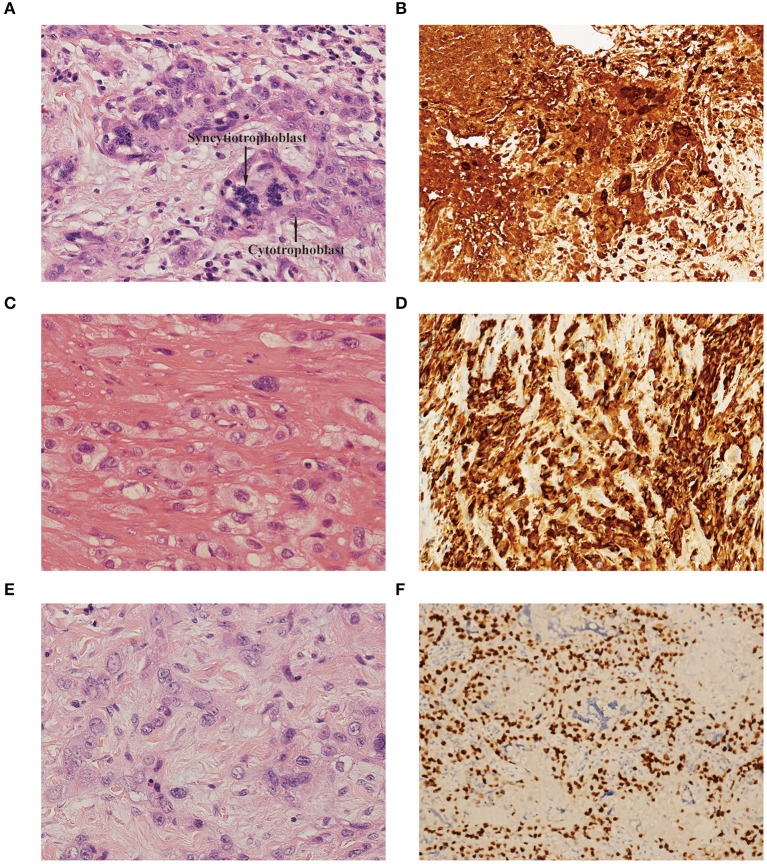
**(A)** Choriocarcinoma component showed biphasic pattern of mononuclear cytotrophoblast and multinuclear syncytiotrophoblast [hematoxylin-eosin (H&E), original magnification×300]. **(B)** Immunohistochemistry showed positive staining for β-hCG in choriocarcinoma (original magnification×150). **(C)** PSTT component showed cords and sheets of monomorphic intermediate trophoblast with pleomorphic nuclei. The tumor cells dissected and separated the smooth muscle bundles. (H&E, original magnification×300). **(D)** Immunohistochemistry showed positive staining for hPL in intermediate trophoblast of PSTT (original magnification×150). **(E)** ETT component showed nests and cords of uniform mononucleate intermediate trophoblastic cells with intercellular eosinophilic hyaline or fibrillar material (H&E, original magnification×300). **(F)** Immunohistochemistry showed positive staining for P63 in intermediate trophoblast of ETT (original magnification×150). PSTT, placental site trophoblastic tumor; ETT, epithelioid trophoblastic tumor; hCG, human chorionic gonadotropin; hPL, human placental lactogen.

In our retrospective study, choriocarcinoma coexisted with ETT and PSTT in 8 patients each. A variety of histopathological features allowed us to differentiate the components of choriocarcinoma, PSTT, and ETT. Microscopically, the choriocarcinoma component showed a typically biphasic pattern with mononuclear cytotrophoblast and multinuclear syncytiotrophoblast (Figure 1A). Immunohistochemical staining for β-hCG revealed positive staining in this component (Figure 1B). PSTT was composed of mononuclear trophoblastic cells forming cords and sheets; the tumor cells extensively infiltrated the endometrium and deeply penetrated the uterine wall (Figure 1C). Immunohistochemistry showed positive staining for human placental lactogen (hPL) in intermediate trophoblast of PSTT (Figure 1D). ETT was composed of a relatively uniform population of mononuclear intermediate trophoblastic cells arranged in nests, cords, or masses. These tumor cells contained round nuclei and eosinophilic or clear cytoplasm, and also exhibited high mitotic activity (Figure 1E). Immunohistochemistry showed positive staining for P63 in intermediate trophoblast of ETT (Figure 1F).

### Treatment

All our patients with mixed GTN received chemotherapy; 14 patients underwent surgery combined with chemotherapy while the remaining 2 received only chemotherapy because of their desire to preserve fertility. The most common chemotherapy regimens included: floxuridine, actinomycin-D, etoposide, and vincristine (FAEV); etoposide, methotrexate, actinomycin-D/cyclophosphamide, and vincristine (EMA/CO); and etoposide, methotrexate, actinomycin-D/etoposide, and cisplatin (EMA/EP). Surgeries consisted of simple abdominal or laparoscopic hysterectomies (*n* = 10), laparoscopic radical hysterectomy (*n* = 1), resection of uterine lesion (*n* = 1) and pulmonary lobectomies (*n* = 3). Detailed information regarding the patients' treatments are listed in Table 1.

### Outcomes

All patients were followed via telephone interviews or at clinics. The median follow-up time was 21 months (range, 1–82 months). Fifteen patients (93.8%) achieved CR after treatment, 5 (33.3%) of whom relapsed within 3 to 9 months; meanwhile, 1 patient died of disease progression. Among the 5 patients who had relapses, 2 (#9 and #12) experienced CR again after chemotherapy and pulmonary lobectomy; 2 (#11 and #16) received immune checkpoint inhibitor (pembrolizumab) treatment after relapse and 1 of them (#11) re-achieved CR; the other 1 (#15) refused to undergo pulmonary lobectomy and was lost to follow-up. Four of the 5 relapsed patients had extrauterine metastases before treatment and 3 of them were resistant to chemotherapy. None of the 2 patients who underwent fertility-sparing treatments experienced a relapse.

## Discussion

Mixed GTN is a rare occurrence that refers to the coexistence of choriocarcinoma and/or PSTT and/or ETT. To this date, such tumors have mainly been described in individual case reports; hence, information about them is relatively scarce, which complicates their diagnosis and management. For this reason, we conducted this retrospective study and literature review to attain a better understanding of mixed GTNs.

Aside from the 16 patients in our retrospective study, 18 other patients with mixed GTN have previously been reported (7–19). The clinical characteristics, diagnoses, and treatments of patients with mixed GTN reported in the literatures are summarized in Table 2. The majority had a choriocarcinoma component, although 3 patients had ETT and PSTT without choriocarcinoma. The most common symptom was abnormal vaginal bleeding. Sixteen of the 18 patients had elevated serum hCG levels, including 6 with levels >10,000 IU/L. The interval between the last pregnancy and commencing treatment ranged from 14 weeks to 38 years. The initial diagnoses of these mixed GTNs varied; none were diagnosed correctly at initial presentation. All 3 patients with mixed PSTT and ETT underwent hysterectomy alone, and none relapsed. Among the 15 patients with choriocarcinoma mixed with ITT, 12 received chemotherapy combined with surgery that included hysterectomy (*n* = 10), uterine horn resection (*n* = 1), and pulmonary lobectomy (*n* = 1); 2 patients underwent only hysterectomy without chemotherapy, and 1 with extensive metastases received only chemotherapy. Among the 15 patients whose survival information was available, 14 patients achieved CR and 1 patient died of disease progression after treatment. After a median follow-up of 45 months (range, 1–238 months) for the 14 patients, 10 were alive without disease; 4 relapsed after remission and yet were alive after salvage treatment.

**Table 2:** Characteristics, diagnoses, and treatments of patients with mixed gestational trophoblastic neoplasia reported in the literature.

**Author**	**Age**	**Symptoms**	**Antecedent pregnancy**	**Pretreatment hCG (IU/L)**	**FIGO stage**	**Initial presumptive Diagnosis**	**Diagnostic method**	**Pathological diagnosis**	**Treatment**	**Relapse**
Shih and Kurman (7) (case 8)	15	Abnormal vaginal bleeding	Hydatidiform mole	NA	I	NA	NA	ETT/PSTT	Hysterectomy	No
Shih and Kurman (7) (case 9)	39	Abnormal vaginal bleeding	Term	2,304	NA	NA	NA	ETT/PSTT/CC	Hysterectomy	No
Shih and Kurman (7) (case 14)	42	Radiologic finding of lung mass	Term	1,300	III	NA	NA	ETT/CC	Pulmonary lobectomy followed by MEA	NA
Knox et al. (8)	20	Abnormal vaginal bleeding	NA	106,153	III	Ectopic pregnancy	D&C	ETT/CC	5 cycles of EMA/CO, TAH, and 3 cycles of EMA/CO	Yes
Ramondetta et al. (9)	60	Cough, hemoptysis	Term	NA (381,561 after operation)	IV	Uterine sarcoma	Hysterectomy	ETT/CC	TAH+BSO, PLND, PALND and omental biopsy followed by 9 cycles of EMA/CO	Yes
Baergen et al. (10) (Case 12710)	30	NA	Abortion	11,000	I	NA	D&C	PSTT/CC	Hysterectomy, and chemotherapy	No
Baergen et al. (10) (case 13094)	28	NA	NA	22,000	IV	NA	D&C	PSTT/CC	Chemotherapy	Died
Cole et al. (11)	52	Vaginal bleeding and vaginal discharge	NA	4,036	III	Malignant mixed mullerian tumor	Hysterectomy	PSTT/ETT/CC	TAH+BSO, then radiation and 3cycles of EMA/CO	Yes
Luk and Friedlander (12)	41	Menorrhagia	NA	NA (2,233 after operation)	I	Fibroids	Hysterectomy	ETT/CC	TAH+BSO, followed by 5 cycles of EMA/EP	No
Sung et al. (13)	32	Abnormal vaginal bleeding	Abortion	313	I	Choriocarcinoma	Hysterectomy	ETT/CC	1 cycle of MTX, 3 cycles of Act-D, 4 cycles of EMA/CO, 4 cycles of EMA/EP, then TAH+LSO	NA
Chen et al. (14)	41	Abnormal vaginal bleeding	Term	1	I	Endometrial hyperplasia	Hysterectomy	ETT/PSTT	TAH	No
Gari (15)	29	Abnormal vaginal bleeding	Term	14,889	III	Retained product of conception	D&C	PSTT/CC	TAH + vaginal mass resection, followed by EMA/CO	No
Imamura et al. (16)	32	Secondary amenorrhea	Ectopic pregnancy	93,820	I	Retained products from ectopic pregnancy	Uterine horn resection	ETT/CC	Laparoscopic uterine horn resection followed by 6 cycles of MEA	No
Zhang et al. (17)	34	Pelvic mass	Term	NA (4,552 after operation)	NA	Leiomyoma	Laparoscopic myomectomy	ETT/PSTT	Hysterectomy	NA
Akakpo et al. (18)	35	Abnormal vaginal bleeding	Abortion	36,900	I	Incomplete miscarriage	D&C	ETT/CC	TAH+BS	Yes
Tse et al. (19) (patient C)	50	Rise of hCG	Hydatidiform mole	192.2	I	Recurrent GTN	Hysterectomy	ETT/CC	5 cycles of CHAMOC, followed by hysterectomy	No
Tse et al. (19) (patient D)	34	Abnormal vaginal bleeding	Term	1,400	I	Ectopic pregnancy and recurrent GTN	Hysterectomy	ETT/CC	16 cycles of EMA/CO, then hysterectomy	No
Tse et al. (19) (patient E)	33	Abnormal vaginal bleeding	Miscarriage	3,139	I	Cornual pregnancy	Resection of tumor	ETT/CC	TAH, followed by 2 cycles of CHAMOC	No

*CC, choriocarcinoma; PSTT, placental site trophoblastic tumor; ETT, epithelioid trophoblastic tumor; D&C, dilation and curettage; Act-D, actinomycin-D; MTX, methotrexate; MEA, methotrexate, etoposide and actinomycin-D; EMA/CO, etoposide, methotrexate, actinomycin-D/cyclophosphamide, vincristine; EMA/EP, etoposide, methotrexate, actinomycin-D/etoposide, cisplatin; CHAMOC, cyclophosphamide, hydroxyurea, actinomycin-D, methotrexate with folinic acid, and vincristine; TAH, total abdominal hysterectomy; BS, bilateral salpingectomy; BSO, bilateral salpingo-oophorectomy; LSO, left salpingo-oophorectomy; PLND, pelvic lymph node dissection; PALND, para-aortic lymph node dissection; NA, not available*.

PSTT and ETT are derived from intermediate trophoblast, therefore, hCG levels in patients with these 2 rare types of GTN are often normal or only mildly elevated. Horowitz et al. reported that hCG levels were <1,000 IU/L in most patients with PSTT and ETT, while high hCG levels (>10,000 IU/L) were often found in patients with choriocarcinoma (3, 20). Among previously reported patients with mixed GTN as well as those in our series, the majority had markedly elevated hCG levels (>10,000 IU/L); such patients are more likely to be suspected of having choriocarcinoma on initial clinical presentation before pathological evidence becomes available. However, owing to the coexistence of ITT with choriocarcinoma, they are unlikely to be as sensitive to chemotherapy. In our cohort, 7 patients with a clinical diagnosis of choriocarcinoma were ultimately found to be chemoresistant, following which coexisting ITT was found with choriocarcinoma on pathological samples post-surgery. Based on these results, we conclude that histologic evaluation is necessary for choriocarcinomas that are resistant to chemotherapy. Pathologists specializing in gynecological oncology should review these specimens carefully to avoid missing any coexisting ITT component that may be present. Furthermore, among choriocarcinomas mixed with ITT, sensitivity to chemotherapy might be associated with the ratio of syncytiotrophoblast/cytotrophoblast-to-intermediate trophoblast (9). Therefore, it is recommended that pathologists investigate the proportions of different mixed GTN components in order to determine whether the clinical behaviors of these tumors are governed by the dominant component.

Given the chemoresistant nature of PSTT and ETT, surgery is the primary treatment modality. Previous studies demonstrated that patients with uterine-confined PSTT or ETT can be cured by primary hysterectomy alone without chemotherapy (3, 5, 6, 21). In our series, the majority of patients with mixed GTN underwent surgeries that included hysterectomy and pulmonary lobectomy. Only 2 patients received chemotherapy alone to preserve fertility, none of whom experienced relapse. The favorable responses to chemotherapy in these 2 patients may be related to the fact that choriocarcinoma was the predominant component. Moreover, chemotherapy should be combined with surgery for patients with choriocarcinoma coexisting with ITT, although adjuvant chemotherapy appears to provide no benefit for patients with uterine-confined tumors composed purely of PSTT or ETT (3). Most patients with mixed GTN usually have high hCG levels owing to the choriocarcinoma component. Feng and Xiang suggested that hCG should be maintained at low levels before surgery to achieve good outcomes (22). Therefore, 13 of the 14 patients in our cohort who underwent surgery received chemotherapy beforehand to lower preoperative hCG levels to the greatest extent possible. Hence, we recommend that surgery for patients with choriocarcinoma coexisting with ITT should be performed after preoperative hCG levels have been maintained at their lowest possible values post-chemotherapy.

In terms of prognosis, 33.3% of patients with mixed GTN in our cohort experienced relapse; this rate appeared to be much higher than that of patients with pure choriocarcinoma or ITT. However, it is difficult to draw the conclusion that the prognoses of patients with mixed GTN are necessarily poor owing to the small sample size in our study. Therefore, more international cooperation that can yield larger studies is required to attain a better understanding of this entity.

Almost all reported mixed GTNs comprised choriocarcinoma and ITT; the exceptions were 3 previously reported patients who had only PSTT and ETT (7, 14, 17). The pathogenesis of mixed GTN is poorly understood. Mao et al. proposed that choriocarcinoma is the most primitive trophoblastic tumor, and is composed of varied amounts of cytotrophoblast, syncytiotrophoblast, and intermediate trophoblast. In contrast, PSTT and ETT are relatively more differentiated. PSTT involves a neoplastic cytotrophoblast differentiating into intermediate trophoblastic cells at the site of implantation, while ETT involves the neoplastic cytotrophoblast differentiating into chorionic-type intermediate trophoblastic cells (23, 24). The model may explain coexistence of mixed GTN. Furthermore, in our series, some post-chemotherapy choriocarcinomas were found to be coexisting with ITT postoperatively. Based on Shih and Kurman's hypothesis (25), it is speculated that multiple courses of chemotherapy may have destroyed most sensitive choriocarcinoma tumor cells; the remaining cells differentiated into intermediate trophoblastic cells that were refractory to chemotherapy. Lu et al. also reported that 4 chemoresistant GTN patients were found to have coexisting ETT postoperatively (26), which further supports the notion that ETT developed from a preexisting choriocarcinoma after chemotherapy.

Our study had certain limitations. Given the rarity of GTNs, we were only able to conduct a retrospective study. Furthermore, it was difficult to draw conclusions regarding the prognoses of patients with mixed GTN owing to the small sample size. However, to the best of our knowledge, our study comprised the largest sample size investigated to date, and we also reviewed all previously reported patients with mixed GTN; hence, our study ought to provide valuable information toward understanding this entity.

In conclusion, the coexistence of choriocarcinoma and/or PSTT and/or ETT is rare, and such patients are difficult to diagnose correctly at initial presentation. On one hand, the presence of coexisting ITT component should be considered in patients with refractory chemoresistant choriocarcinoma, and histological evaluation should be performed. On the other hand, coexisting choriocarcinoma should be suspected in ITT patients with high hCG levels; pathologists should review the specimens carefully so as not to miss the possibility of coexisting choriocarcinoma. Surgery combined with chemotherapy is the best treatment regimen for choriocarcinoma coexisting with ITT.

## Data Availability Statement

The raw data supporting the conclusions of this manuscript will be made available by the authors, without undue reservation, to any qualified researcher.

## Ethics Statement

The studies involving human participants were reviewed and approved by The Ethics Committees of Peking Union Medical College Hospital and The Ethics Committees of The Second Xiangya Hospital of Central South University. The patients/participants provided their written informed consent to participate in this study. Written informed consent was obtained from the individual(s) for the publication of any potentially identifiable images or data included in this article.

## Author Contributions

YK and YX contributed to the conception and design of this study. YK, GT, JY, and XW contributed to data acquisition. YK and WW contributed to data interpretation and analysis. WW and YX contributed to study supervision. YK and LZ contributed to manuscript editing. All authors contributed to manuscript review.

### Conflict of Interest

The authors declare that the research was conducted in the absence of any commercial or financial relationships that could be construed as a potential conflict of interest.
